# Diseases in Patients Coming to a Sleep Center with Symptoms Related to Restless Legs Syndrome

**DOI:** 10.1371/journal.pone.0071499

**Published:** 2013-08-19

**Authors:** Shih-Wei Lin, Yen-Lung Chen, Kuo-Chin Kao, Cheng-Ta Yang, Li-Pang Chuang, Yu-Ting Chou, Szu-Chia Lai, Rou-Shayn Chen, Ning-Hung Chen

**Affiliations:** 1 Sleep Center, Pulmonary and Critical Care Medicine, Chang Gung Memorial Hospital, Tayuan, Taiwan; 2 Center for Traditional Chinese Medicine, Chang Gung Memorial Hospital, Chang Gung University, Taoyuan, Taiwan; 3 Department of Thoracic Medicine and Respiratory Therapy, Chang Gung Memorial Hospital, Taoyuan, Taiwan; 4 Sleep Center, Movement Disorder Section, Department of Neurology, Chang Gung Memorial Hospital, Taoyuan, Taiwan; 5 Graduate Institute of Traditional Chinese Medicine, Chang Gung University, Taoyuan, Taiwan; 6 Graduate Institute of Clinical Medical Sciences, Chang Gung University, Taoyuan, Taiwan; 7 Department of Respiratory Therapy, Chang Gung University, Taoyuan, Taiwan; 8 Pulmonary and Critical Care Medicine, Chang Gung Memorial Hospital, Chiayi, Taiwan; Penn State Hershey Medical Center, United States of America

## Abstract

**Study Objective:**

To explore the profile of patients who visit a sleep center with symptoms that fulfill the four essential criteria for restless legs syndrome (RLS).

**Design:**

A prospective study.

**Setting:**

Outpatients from one sleep disorders clinic in Taiwan.

**Participants:**

1,200 consecutive patients visit sleep disorders clinic with any sleep complaints.

**Interventions:**

After completing a history and physical examination, all participants answered the RLS questionnaire. Subjects who fulfilled the four essential criteria for RLS were referred to a special clinic. A work-up including blood tests, polysomnography, and specialized neurological tests etc. was performed to make the final diagnosis.

**Measurements and Results:**

A total of 1,185 participants were enrolled, and, of these, 131(11.1%) fulfilled the four essential criteria for RLS, and 121 completed the supplemental work-up. Their mean age was 47.6±13.3 and 52.9% were male. Insomnia and snoring were the most common chief complaints. Obstructive sleep apnea syndrome and other diseases were found in 103 patients. Only 18 (14.9%) patients had no comorbid condition and were diagnosed with primary RLS.

**Conclusions:**

Symptoms of RLS are common in patients with sleep complaints. Even in a sleep clinic, using a questionnaire approach for identification of RLS has a low positive predictive value. Clinicians should pay attention to the limitations of the 4-item questionnaire in diagnosis of RLS and also the importance of a careful differential diagnosis to identify possible secondary causes of RLS.

## Introduction

Restless legs syndrome (RLS) is a common sensorimotor disorder and is characterized by an urge to move associated with dysesthesia in the legs, worse during periods of rest or inactivity, partially or totally relieved by movement, and worse near evening or at night [1]. RLS is an important sleep disorder with a reported prevalence around 4% to 29% [2]. In our previous study, the prevalence was 1.57% in Taiwan [3]. This was much lower than that reported in other countries. The prevalence in other Asian countries was also low, from 0.9% to 3.9% [4]–[7]. The reason for the lower prevalence of RLS in Asian countries is not clear.

The diagnostic criteria for RLS were developed in 1995, and updated in 2003 by the International Restless Legs Syndrome Study Group (IRLSSG) [1]. The diagnosis is based on clinical evaluation and no definitive biomarker is available. Objective information including excessive periodic leg movements, positive response to dopaminergic agent, family history of RLS or findings of a neurological test are considered both supportive for RLS diagnosis and important for decisions on differential diagnoses [8]. The four essential diagnostic criteria have provided the basis for most questionnaires used in epidemiological studies. This can lead to rough diagnosis without careful consideration of differential diagnosis. This is a problem since the questionnaire has a positive predictive value about 45–60% for RLS [9], [10]. In Asian population, the lower prevalence of clinically significant RLS would reduce the positive predictive value to below 50%.

Most patients with RLS complain of either insomnia or sleepiness and these are also the most frequent complaints in patients who visit sleep centers [11]. RLS will be easily under-diagnosed by sleep physicians due to the nonspecific symptoms, and ignored by patients come without knowledge about RLS. The diagnosis of RLS also needs to exclude many different diseases such as iron deficiency, peripheral neuropathy, renal dysfunction and medication-related effects. A study reported by Allen *et al.* assessed more strictly primary RLS [12]. They gave a conservative American population prevalence estimate of 2.4% for primary RLS and 1.5% for primary RLS suffers (symptoms >2/week with moderate-to-severe distress). We assume that the patients in Taiwanese population sought help at sleep clinic would be considered to have clinically significant RLS if they had RLS at all. When RLS is moderately severe, it produces high personal and social costs, including a significant workplace productivity loss [12].

Thus, the diagnosis of primary RLS in patients who visit sleep centers is a challenge. It would be helpful to know the differences between patients with primary RLS, and those with secondary RLS that may share the same symptoms as RLS. In this study, our aim sough to explore the profile of patients whose symptoms fulfilled 4-item RLS diagnostic criteria, and the associated diseases producing symptoms that meet the RLS diagnosis criteria in a Taiwan sleep center.

## Methods

Consecutive patients who visited the Sleep Clinic of Chang Gung Memorial Hospital in Taiwan with any sleep related complaint from November 2007 to October 2008 were invited to participate in the study. Those who were under 18 years old or illiterate were excluded. After a complete clinical history and physical examination, the participants were requested to answer a self-administered questionnaire survey about RLS. The questionnaire included four questions based on the essential diagnostic criteria for RLS as set by the IRLSSG in 2003 [1]. The four questions were: 1. “Do you have an urge to move, usually accompanied or caused by uncomfortable and unpleasant sensations in the legs?” 2. “Does the urge to move or unpleasant sensations begin or worsen during periods of rest or inactivity such as lying or sitting?” 3. “ Are the urge to move or unpleasant sensations partially or totally relieved by movement, such as walking or stretching, at least as long as the activity continues?” 4. “Are the urge to move or unpleasant sensations worse in the evening or at night than during the day or do they occur only in the evening or at night?” The Chinese version of four-item questionnaire was used in prior published researches conducted by our team [13], [14].

Subjects who fulfilled all four criteria were referred to a specialist who owned the boards of both neurology and sleep medicine. The neurologist followed IRLSSG criteria, and also evaluated the presence of supportive clinical features, including positive family history of RLS, response to treatment, and periodic limb movements, to resolve any diagnostic uncertainty. According to the diagnosis procedure suggested, [15] blood tests including hemoglobin/hematocrit, iron profile, renal function, blood sugar and thyroid function were scheduled. Overnight polysomnography was done for suspected sleep-disordered breathing or periodic limbs movement syndrome. Medication was carefully reviewed for any provocation agents. Specialized neurological tests, including somatosensory evoked potential and nerve conduction velocity (NCV) testing, were arranged for suspected neuropathy. The final diagnosis was made after all diagnostic procedures were completed.

The differences between groups of subjects were analyzed with chi-squared statistics for categorical variables and with t-tests for quantitative variables. The statistical significance was defined as *p*<0.05.

### Ethics statement

Written informed consent was obtained from all participants. This study was approved by the Chang Gung Medical Foundation Institutional Review Board.

## Results

A total of 1,200 patients were enrolled in the study. Of these, 15 were excluded due to incomplete data and 131 (11.1%) fulfilled all 4 criteria for RLS. Ten of these 131 participants were lost to follow-up, and 121 (10.2%) completed further evaluation. Of these 121 patients, 64 were male and 57 were female. The mean age was 47.6±13.3 and 66 of the 121 (54.5%) were between 41 and 60 years of age. The chief complaints included insomnia, snoring or witnessed apnea, discomfort in the limbs, daytime sleepiness, chest pain or tightness, and low back pain. Insomnia (difficulty in initiating or maintaining sleep and poor sleep quality) was the most common complaint. (Table 1) After completing the supplemental work-up, 103 patients were diagnosed as having obstructive sleep apnea syndrome (OSAS) or other diseases (end-stage renal disease, peripheral neuropathy, iron deficiency, thyroid disorders, Parkinson's disease, rheumatoid arthritis). (Table 2) More than 50% of the patients with other diseases were OSAS. 10 of 15 patients with iron deficiency had no anemia. Only 18 (14.9%) patients had no comorbid condition and were diagnosed with primary RLS. Of these 18 patients with primary RLS, the female patients were predominant. (Table 1) The leading chief complaints were insomnia and snoring, but only 11.1% had the chief complaint of limb discomfort. (Table 1) The results of blood tests showed significant difference between male and female patients. ( Table S1) Low serum ferritin level was found in none of the male patients and only 5 of the female patients.

**Table 1:** Demographics of patients who fulfilled all the criteria for restless leg syndrome.

	Primary RLS	Other diseases	Total	*p* value
	(*n* = 18) (%)	(*n* = 103) (%)	(n = 121) (%)	
**Gender**				0.021*
Male	5 (27.8)	59 (57.3)	64 (52.9)	
Female	13 (72.2)	44 (42.7)	57 (47.1)	
**Age**				0.539
21 to 30	3 (16.7)	7 (6.8)	10 (8.3)	
31 to 40	2 (11.1)	22 (21.4)	24 (19.8)	
41 to 50	5 (27.87)	29 (28.2)	34 (28.1)	
51 to 60	6 (33.3)	26 (25.2)	32 (26.4)	
61 to 70	2 (11.1)	13 (12.6)	15 (12.4)	
Above 70	0 (0.0)	6 (5.8)	6 (5.0)	
**Chief complaint**				
Insomnia^+^	12 (66.7)	46 (44.7)	58 (47.9)	0.047*
Snoring or witnessed apnea	2 (11.1)	34 (33.0)	36 (29.8)	0.039*
Discomfort on limbs	2 (11.1)	16 (15.5)	18 (14.9)	0.269
Daytime sleepiness	1 (5.6)	3 (2.9)	4 (3.3)	0.375
Chest pain or tightness	1 (5.6)	3 (2.9)	4 (3.3)	0.375
Low back pain	0 (0.0)	1 (1.0)	1 (0.8)	-

+Insomnia including dreamy sleep, difficulty in initiating or maintaining sleeping and poor sleep quality.

* statistical significance between groups.

**Table 2:** Diseases that Fulfilled the Criteria for RLS in Chang Gung Sleep Center.

	*n* (%)
Iron deficiency	15 (12.4%)
With anemia	5 (4.1%)
Without anemia	10 (8.3%)
Thyroid dysfunction	4 (3.3%)
Hyperthyroidism	2 (1.7%)
Hypothyroidism	2 (1.7%)
Obstructive sleep apnea	67 (55.4%)
Neuropathy	35 (28.9%)
Peripheral neuropathy	17 (14.0%)
Polyneuropathy	8 (6.6%)
Radiculopathy	3 (2.5%)
Myelopathy	7 (5.8%)
End-stage renal disease	9 (7.4%)
Parkinson's disease	5 (4.1%)
Rheumatic disease	2 (1.7%)
Medication-related (antidepressants)	2 (1.7%)

## Discussion

The urge to move and discomfort in the legs are the major symptoms of RLS. In clinical practice, these symptoms are difficult to clearly distinguish from other conditions such as neurological disorders, rheumatoid arthritis or sleep disorders. Thus, the symptoms of RLS may be ignored by patients and RLS may be under-diagnosed by physicians. Screening a primary care population by using a RLS diagnostic questionnaire, Nichols *et al*. reported that the prevalence of RLS was 24.0% [16]. In our study, 11.1% of patients who visited the sleep center fulfilled the criteria for RLS. This was much lower than studies in Caucasian populations [17], [18]. Only 1.5% (18/1,185) of the total participants was diagnosed as primary RLS. This prevalence rate was similar to that in our previous report which was 1.57% of the general Taiwanese population, [3] and differed from 2.4% in American population [12] as our estimation.

Female predominance was noted in patients with primary RLS who visited our sleep center. This observation was also noted in other surveys [9], [17], [19] but in our previous report of a general population,^3^ gender showed no significant difference. Differences in medical behavioral or sampling differences could be possible explanations for this. Insomnia was the most significant complaint by those who were finally diagnosed with primary RLS whereas daytime sleepiness seemed not to be significant.

Most of the patients who fulfilled 4-item essential diagnostic criteria for RLS had diseases other than RLS (84.3%). These other diseases included OSAS, iron deficiency, thyroid dysfunction, neuropathy, end-stage renal disease, Parkinson's disease, rheumatoid arthritis, and medication-related effects. A study reported by Allen *et al.* [20] surveyed a large sample (n = 10,564) of primary medical care patients in western Europe and revealed that 7.6% of these patients had a positive result for RLS on the basis of the screening questionnaire, and they estimated the prevalence of RLS to be 3.5% as diagnosed by well-trained primary care physicians after careful evaluation. In the absence of validated, universally applicable objective measures, the criteria for the diagnosis of RLS remain dependent upon the clinician's judgment. The diagnosis may be particularly challenging in the patients with comorbid conditions that mimic symptoms of RLS.

Among our 121 patients with RLS symptoms, 47.9% had a chief complaint of insomnia. RLS patients have reduced quality of sleep due to RLS related symptoms [20]. Insomnia and snoring are also the most frequent complaints of patients who visit sleep clinics. Therefore, sleep specialists should consider RLS when managing all patients in the sleep clinic.

Snoring is the major complaint of patients who come to a sleep center and who fulfill all criteria for RLS of other diseases. OSAS was the most frequent condition diagnosed in patients who visited our sleep center and our results were similar to those in a previous report that RLS commonly occurred in patients with sleep apnea [21]. Delgado Rodrigues *et al*. reported that RLS showed a favorable response to continuous positive airway pressure treatment in patients with OSAS and RLS [22]. Polysomnography is recommended for patients when a sleep center is charged by a neurologist with ruling out sleep apnea.

According to the recommendation by IRLSSG, evaluations of serum ferritin levels and percent iron saturation are suggested as part of the medical evaluation for RLS. Iron deficiency is one of the factors that may exacerbate the symptoms of RLS. A low-normal serum ferritin level was related to increased severity of RLS and may be associated with an increased risk of the occurrence of RLS even in patients without anemia [23], [24]. The iron status of our participants showed in table S1. The results of most of the patients (87.4%) were within normal limit. The possible reason to explain this result may be patient population. The study reported by O'Keeffe *et al*. focus on the elderly. The cases in the study reported by Sun *at el.* were not on iron or medications that might reduce the RLS symptoms. Our neurologist followed the criteria updated in 2003 strictly including patients with iron deficiency were identified as secondary RLS only if the RLS is not corrected with iron treatment.

IRLSSG diagnostic criteria for RLS were revised in 2011, and the fifth criterion was added to exclude RLS mimics. (http://irlssg.org/diagnostic-criteria/) These conditions were commonly confused with RLS. Although our study was conducted without the fifth criterion, our principle and procedure did not conflict IRLSSG diagnostic criteria for RLS revised in 2011. Our result confirmed the limitation of 4-item RLS questionnaire in the differentiation of secondary RLS. The Cambridge-Hopkins questionnaire was reported by Allen *et al.*, [25] and reduced error mostly in RLS mimics.

In summary, symptoms of RLS are common in patients with sleep complaints. The study shows that even in a sleep clinic using a four-item questionnaire approach for identification of RLS has a low positive predictive value. Clinicians should pay attention to the limitations of the 4-item questionnaire in diagnosis of RLS and also the importance of a careful differential diagnosis to identify possible secondary causes of RLS.

## Supporting Information

Table S1 **The results of blood tests of 121 patients fulfilled four essential diagnostic criteria for RLS.**
(DOC)
