# Supplementary material for: Diseases in Patients Coming to a Sleep Center with Symptoms Related to Restless Legs Syndrome
Source: PLoS One. 2013 Aug 19;8(8):e71499. doi: 10.1371/journal.pone.0071499 (PMC3747238; doi:10.1371/journal.pone.0071499)
Supplement: Table S1 — The results of blood tests of 121 patients fulfilled four essential diagnostic criteria for RLS. (DOC) [file pone.0071499.s001.doc]

Table S1. The results of blood tests of 121 patients fulfilled four essential diagnostic criteria for RLS

|  | total | male | female | *p* value |
| --- | --- | --- | --- | --- |
|  |  |  |  |  |
| Hb(g/dL) | 13.8±1.9 | 15.1±1.5 | 12.7±1.6 | 0 |
| serum iron(μg/dL) | 97.5±41.0 | 107.0±39.0 | 90.1±41.6 | 0.09 |
| ferritin(ng/mL) | 176.7±186.0 | 261.2±165.8 | 121.8±179.4 | 0.002 |
| TIBC(μg/dL) | 325.6±48.6 | 323.6±39.5 | 327.2±55.3 | 0.76 |
| free T4(ng/dL) | 1.8±2.4 | 1.26±0.15 | 2.11±2.97 | 0.54 |
| TSH(μIU/mL) | 6.1±31.6 | 1.78±1.21 | 8.20±38.62 | 0.46 |

Hb, hemoglobin; TIBC, total iron-binding capacity; TSH, thyroid-stimulating hormone

*p*<0.05 denotes statistical significance
